# Is self-generated thought a means of social problem solving?

**DOI:** 10.3389/fpsyg.2013.00962

**Published:** 2013-12-23

**Authors:** Florence J. M. Ruby, Jonathan Smallwood, Jerome Sackur, Tania Singer

**Affiliations:** ^1^Department of Social Neuroscience, Max Planck Institute for Human Cognitive and Brain SciencesLeipzig, Germany; ^2^Laboratoire de Sciences Cognitives and Psycholinguistique, Ecole Normale Supérieure - CNRSParis, France

**Keywords:** self-generated thought, mind-wandering, social problem-solving, thought content, task performance and analysis

## Abstract

Appropriate social problem solving constitutes a critical skill for individuals and may rely on processes important for self-generated thought (SGT). The aim of the current study was to investigate the link between SGT and social problem solving. Using the Means-End Problem Solving task (MEPS), we assessed participants' abilities to resolve daily social problems in terms of overall efficiency and number of relevant means they provided to reach the given solution. Participants also performed a non-demanding choice reaction time task (CRT) and a moderately-demanding working memory task (WM) as a context in which to measure their SGT (assessed via thought sampling). We found that although overall SGT was associated with lower MEPS efficiency, it was also associated with higher relevant means, perhaps because both depend on the capacity to generate cognition that is independent from the hear and now. The specific content of SGT did not differentially predict individual differences in social problem solving, suggesting that the relationship may depend on SGT regardless of its content. In addition, we also found that performance at the WM but not the CRT was linked to overall better MEPS performance, suggesting that individuals good at social processing are also distinguished by their capacity to constrain attention to an external task. Our results provide novel evidence that the capacity for SGT is implicated in the process by which solutions to social problems are generated, although optimal problem solving may be achieved by individuals who display a suitable balance between SGT and cognition derived from perceptual input.

## Introduction

The social world poses many of the most complex problems that we as a species have to solve. Our interactions with other people directly impact upon success in inter-personal relationships and influence many aspects of our lives such as our happiness, our health, and our success in our job and as a family member. The capacity to define and implement the correct strategy in a given social setting is therefore a skill that determines the degree of fit between individuals and their social environment.

Recent work in psychology and cognitive neuroscience has highlighted that in daily life cognition is not always generated from perceptual information. Instead states such as mind-wandering or daydreaming illustrate that humans have the capacity to self-generate thoughts based on previously-stored information rather than events present in the perceptual environment (Smallwood, 2013). Experience sampling studies suggest that such self-generated thoughts (SGT) account for fifty percent of waking thought and content analysis suggests that across cultures these experiences are mainly focused on the self in the future (e.g., Smallwood et al., 2009; Andrews-Hanna et al., 2010; Baird et al., 2011; Iijima and Tanno, 2012; Song and Wang, 2012), implying that these episodes may reflect a form of autobiographical planning (Baird et al., 2011). Despite the high frequency of SGT in daily life, there is no clear consensus on the purpose that these experiences may serve. The current experiment examines whether these states may be related to social problem solving processes.

Successful problem solving depends on two related processes (D'Zurilla and Goldfried, 1971). The first is a capacity to imagine conditions that would allow the agent to move forward on the problem, and the second is a capacity to selectively implement the most effective strategy, a process we will refer to as controlled processing. Although the capacity for controlled processing can arguably be common to both self-generated and perceptually guided thought (Smallwood, 2013) it is possible that the role of imagination necessary for social problem solving could be specifically related to SGT. For example, the ability to imagine the series of steps that allow a social problem to be solved and the ability to self-generate thought both depend on the capacity to imagine events that are unrelated to the present moment (Frith and Frith, 2006; Smallwood et al., 2012). The main goal of the current study was to assess whether individual differences in SGT are related to processes of social problem solving, and if so, whether specific forms of mental content mediate this relation.

In a large cohort of participants social problem solving was assessed using the Means-End Problem Solving (MEPS) task, a validated measure of individuals' abilities to solve common everyday social problems (Platt and Spivack, 1975; Marx et al., 1992; Lyubomirsky and Nolenhoeksema, 1995). The task of the participants is to create a story that would allow her/him to resolve a given problem (e.g., an argument with one's partner, making friends in a new neighborhood). The MEPS is used to assess two distinct aspects of social problem solving: (i) the ability to generate relevant means (RM) i.e., the number of specific steps that the participant provides to solve a given problem; (ii) the overall efficiency of the story, a metric for individuals' capacity to implement an appropriate and efficient strategy (Marx et al., 1992).

As the SGT experience varies depending on the context in which it occurs (for a review, see Smallwood and Andrews-Hanna, 2013), we measured SGT during two cognitive tasks: (a) a moderately-demanding Working Memory (WM) task and (b) a non-demanding Choice Reaction Time task [CRT; for previous use of these tasks see Smallwood et al. (2011a,b, 2013)]. The use of two tasks with variable difficulty allows us to manipulate SGT occurrence and to introduce within-subject variance, as SGT is routinely reduced in the WM task relative to the CRT task. Thought-sampling was used to measure participants' SGT while they performed these tasks. Participants reported the content of their thoughts on a number of dimensions: (i) their relevance to the task being performed, (ii) their temporal focus (future or past), (iii) their social focus (self- or other-related), and (iv) the relative level of detail. Finally, task performance was also measured [response time (RT) and accuracy] and provided a measure of individuals' effectiveness at implementing cognition related to perceptual information.

In this study we were motivated to understand whether patterns of variance in the content of SGT, as well as the conditions under which they arise provide informative information on the psychological nature of different types of thought (Smallwood and Andrews-Hanna, 2013; Smallwood, 2013). The methodological approach used in the current study to describe SGT content is based on our prior published work (Ruby et al., 2013). Using similar probe questions, we applied Principal Component Analysis (PCA) to decompose the probe ratings based on the pattern of covariance between the reports. This allowed us to define different categories of thoughts based on trial-by-trial changes in co-variance. Because we apply unconstrained PCA to decompose our data, this step captures both within-subject and between-subject sources of variance. Using these PCA components as dependent variables in subsequent analyses, we next quantify whether SGT are related to independent variables (such as the task context in which the measure was taken, or features of the individual who made the report). As this technique is relatively novel, we will compare the results obtained using the PCA components with results where rating averages were used in a more standard way. In the discussion we consider in detail the rationale for employing this approach.

The main goal of the current experiment was to assess how social problem solving skills are related to individual differences in SGT. To assess this question we adopted an individual difference analysis and examined the co-variance between different elements of MEPS performance and different types of SGT. One possibility is that SGT, regardless of its content, may be related to performance on the MEPS. This would be the case if all types of SGT predicted MEPS performance in a similar way and would be reflected by a main effect of MEPS. A second possibility is that only SGT with specific content are related to social problem solving. This would be the case if our analysis revealed that only certain types of SGT significantly predict MEPS performance and would be reflected by an interaction between the specific form SGT and performance on the MEPS. For example, as social problem solving is important for anticipating how others may react in the future it is possible that SGT directed toward the future or other people may be stronger predictors of MEPS performance compared to SGT directed toward the self or the past. Finally, our experimental design also allowed us to assess two subsidiary questions: (i) the relation between MEPS and cognitive tasks performance and (ii) the link between SGT and performance on the two cognitive tasks.

## Methods

### Participants

The study was part of a broader research project which was approved by the Ethics Commission of the Medical Faculty of the University of Leipzig under the code 360-10-13122010. 94 right-handed individuals were recruited from the database of the Max Planck Institute for Human Cognitive and Brain Sciences, Leipzig, Germany (Mean age = 29 years, Age range 19–38 years, 49 females). All of them were native German speakers, had normal or corrected-to-normal vision, no history of psychiatric or neurological conditions, and no history of substance abuse. Ten participants were subsequently excluded from the analysis due to extreme scores on the CRT, the WM, or the MEPS [scores were considered as extreme when higher than (Q3 + 1.5 × IQR) or lower than (Q1 – 1.5 × IQR), with Q1 and Q3 the first and third quartiles, and IQR being the Interquartile Range].

### Experimental session

The experimental session lasted 2 h and was divided into three blocks (block order counterbalanced across subjects). Participants were allowed to take short breaks between the blocks if desired. One block consisted of performing the cognitive tasks; another block consisted of performing the MEPS. A number of other tasks were measured during the third block and results have been previously published (Smallwood et al., 2013). All participants gave written consent before the beginning of the experiment and were remunerated at least 16 € for their participation (8 € per hour of participation plus an additional reward according to their performance during a temporal discounting task). E-prime 2 was used for stimulus presentation (Schneider et al., 2002). All statistical analyses were performed using R and results were plotted using the ggplot2 package (Wickham, 2009; R Core Team, 2012).

### Cognitive tasks

Participants performed two sessions of two cognitive tasks: a Choice Reaction Time Task (CRT) and a Working Memory Task [WM; (Smallwood et al., 2009, 2011a,b, 2013)]. Each session lasted 400 s and participants could take a short break between sessions if desired. During the CRT task, participants observed a sequence of black digits on a computer screen while waiting for a target (a colored digit) to appear, at which point they had to indicate the parity of this target (odd or even) with a button push. In the WM task, participants were exposed to the same sequence of black digits, and were intermittently probed with a colored question mark (“?”). When the question mark was presented, participants had to make a button push to indicate the parity of the previous digit. Because the occurrence of the colored question mark is randomly determined, this task requires participants to encode and retain in memory the parity of each non-colored number and make a response only when probed by the question mark. In both tasks, black digits were presented for 1000 ms and colored stimuli were presented for 2000 ms. Events were separated by a fixation cross of random duration (2200, 2800, 3200, or 4400 ms). Targets (or question marks) and non-targets were presented with a ratio of ~1/6. The average number of targets did not differ between CRT and WM tasks (mean target number, CRT task: *M* = 22.5, *SE* = 0.4; WM task: *M* = 22.8, *SE* = 0.5).

During both the CRT and WM tasks, SGT were recorded using thought-sampling (average number of probes: CRT task, *M* = 7.10, *SE* = 0.2; WM task, *M* = 7.07, *SE* = 0.2). Intermittently throughout the tasks, participants were interrupted and asked to rate how much their SGT were (i) unrelated to the current task (i.e., “off-task”); (ii) detailed; (iii) future-focused; (iv) past-focused; (v) self-focused; and (vi) other-focused. Participants used Likert scales to answer the probes [1 to 9, a greater score indicating higher relevance. See Christoff et al. (2009), Mrazek et al. (2012a) for previous uses of this method].

### Means end problem solving task

Social problem solving ability was assessed using a modified version of the Means-End Problem Solving task [MEPS, (Platt and Spivack, 1975; Marx et al., 1992)]. The German version of the task was obtained from Svaldi et al. (2011). Participants read short scenarios presenting a problem (e.g., “you just moved into a new neighborhood and you do not know anybody”) and a solution (e.g., “the story ends when you have several good friends and you feel at home in the new neighborhood”). The task consists of creating a story that would allow the participant to reach the stated solution and therefore solve the problem. Participants are instructed to be as specific as possible, so that other people reading their story would easily understand how the solution was achieved. Following Lyubomirsky and Nolenhoeksema (1995) problems were written using second person pronouns and participants were asked to provide a story based on what they would actually do if they were indeed confronted with such problems. Four different scenarios were used. Each scenario was presented on a computer screen and participants had 4 min to type in a story that would link the stated problem to the solution.

Two independent raters coded each story according to two measures: (i) number of relevant means (RM), representing the number of discrete steps that would allow the participant to get closer to the goal; (ii) Efficiency, representing the global efficiency of the story proposed (how efficient the strategy would actually be at solving the problem) and rated using a 0 to 7 Likert scale. For each participant, a mean Efficiency score and a mean RM score was obtained by averaging the ratings across stories and across raters (Inter-rater reliability Cronbach's α, Efficiency: α = 0.79; for RM: α = 0.79).

## Results

### Descriptive analyses

#### MEPS performance

Measures obtained during the MEPS were similar to previously-reported findings [e.g., (Watkins and Baracaia, 2002)]. Mean efficiency was 3.39 (*SE* = 0.14) and mean RM was 2.68 (*SE* = 0.11). Both measures were highly correlated [Pearson's correlation coefficient (*r* = 0.72, *p* < 0.001)]. Measures of MEPS Efficiency and RM were z-scored prior to performing subsequent analyses.

#### Task performance

The average Error rate for the CRT task was 0.053 (*SE* = 0.006) and for WM task 0.033 (*SE* = 0.005). The average RT was 799 ms (*SE* = 13 ms) for the CRT task and 759 ms (*SE* = 22 ms) for the WM task. Consistent with previous studies [e.g., (Baird et al., 2012)], both error rate and RT for correct responses were lower during the WM compared to the CRT task (paired sample *t*-test, Error rate: *t* = 2.62, *p* = 0.01, RT: *t* = 2.25, *p* < 0.03). This confirms that during the WM task, participants were more focused on the task than during the CRT.

#### Task modulation of SGT

**Figure 1:**
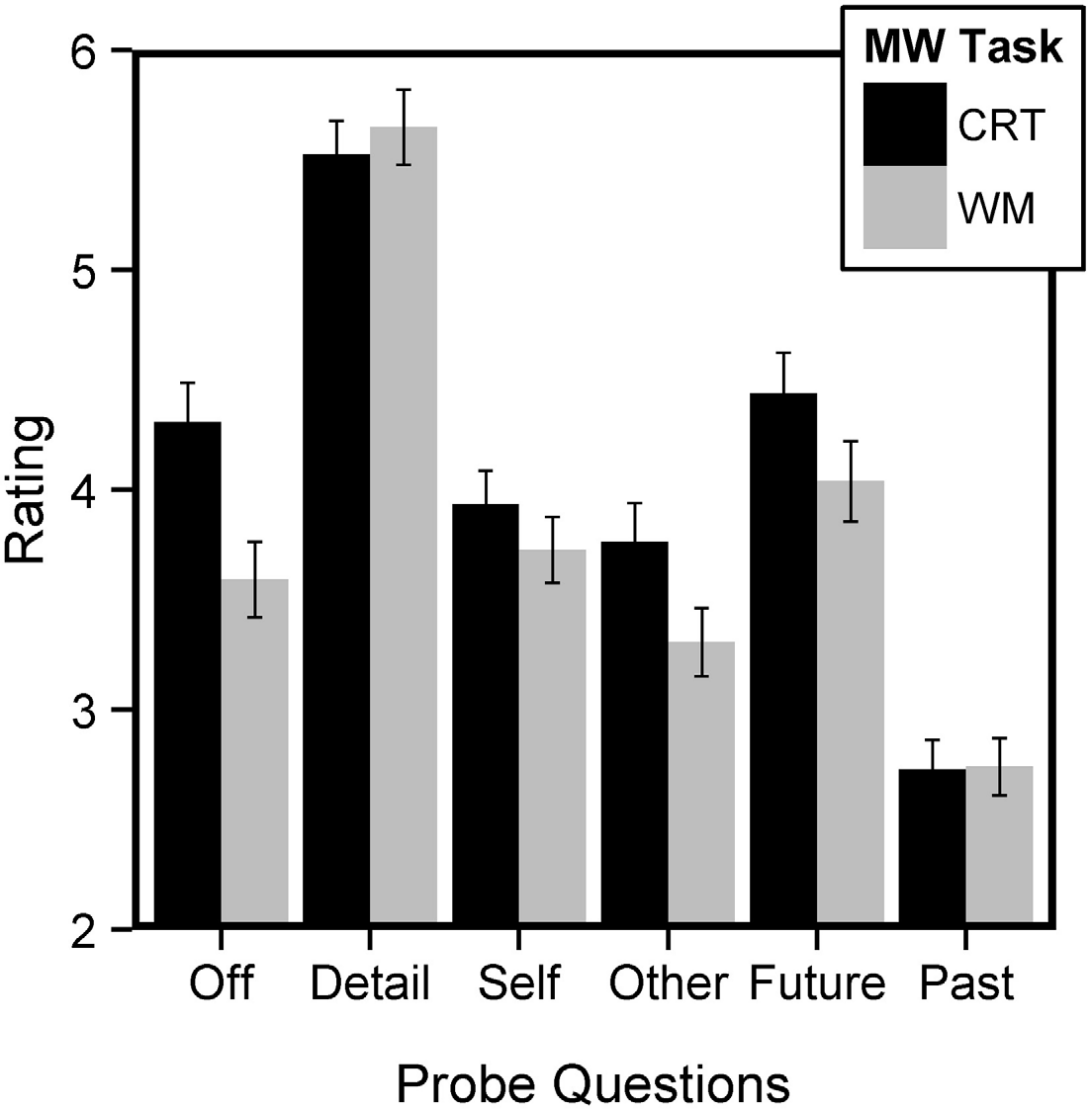
**Mean SGT ratings across mind-wandering (MW) tasks**. SGT in the CRT task were rated as more Off-task, more Self and Other-related, and more Future-related than SGT in the WM task. In addition, SGT were overall more future than past-related.

Off-task ratings were higher in the CRT than the WM task (paired sample *t*-test, *t* = 4.84, *p* < 0.001). Regarding temporal questions, a repeated-measures 2 × 2 ANOVA with Task (CRT, WM) and Probe Question (Future, Past) revealed two main effects and a significant interaction (Task: *F* = 5.13, *p* = 0.03; Probe Question: *F* = 76.54, *p* < 0.001; Interaction: *F* = 5.02, *p* < 0.03). As can be seen on Figure 1, future ratings were higher than past ratings and ratings were higher in the CRT compared to the WM task. The difference in ratings across tasks was especially pronounced for the Future probes. These results replicate previous findings of a future bias of SGT that has now been observed in several different cultures (Smallwood et al., 2009, 2011a; Baird et al., 2011; Iijima and Tanno, 2012; Song and Wang, 2012). A similar ANOVA was performed with social questions (Probe Question: Self, Other). We observed a significant main effect of Task (*F* = 11.38, *p* = 0.001) and a trend for an effect of Probe Question (*F* = 3.01, *p* < 0.09). No significant interaction was obtained (*F* = 1.88, *p* = 0.17). This suggests that social-related ratings are higher during the CRT compared to the WM task (Figure 1). Finally, we found no evidence for a difference of detail ratings across tasks (paired sample *t*-test, *t* = −0.89, *p* > 0.35). Overall, these results suggest that there is significant within-subject variance present in thought-probe ratings across cognitive tasks.

### Link between social problem solving and SGT

#### Decomposition of SGT content

**Figure 2:**
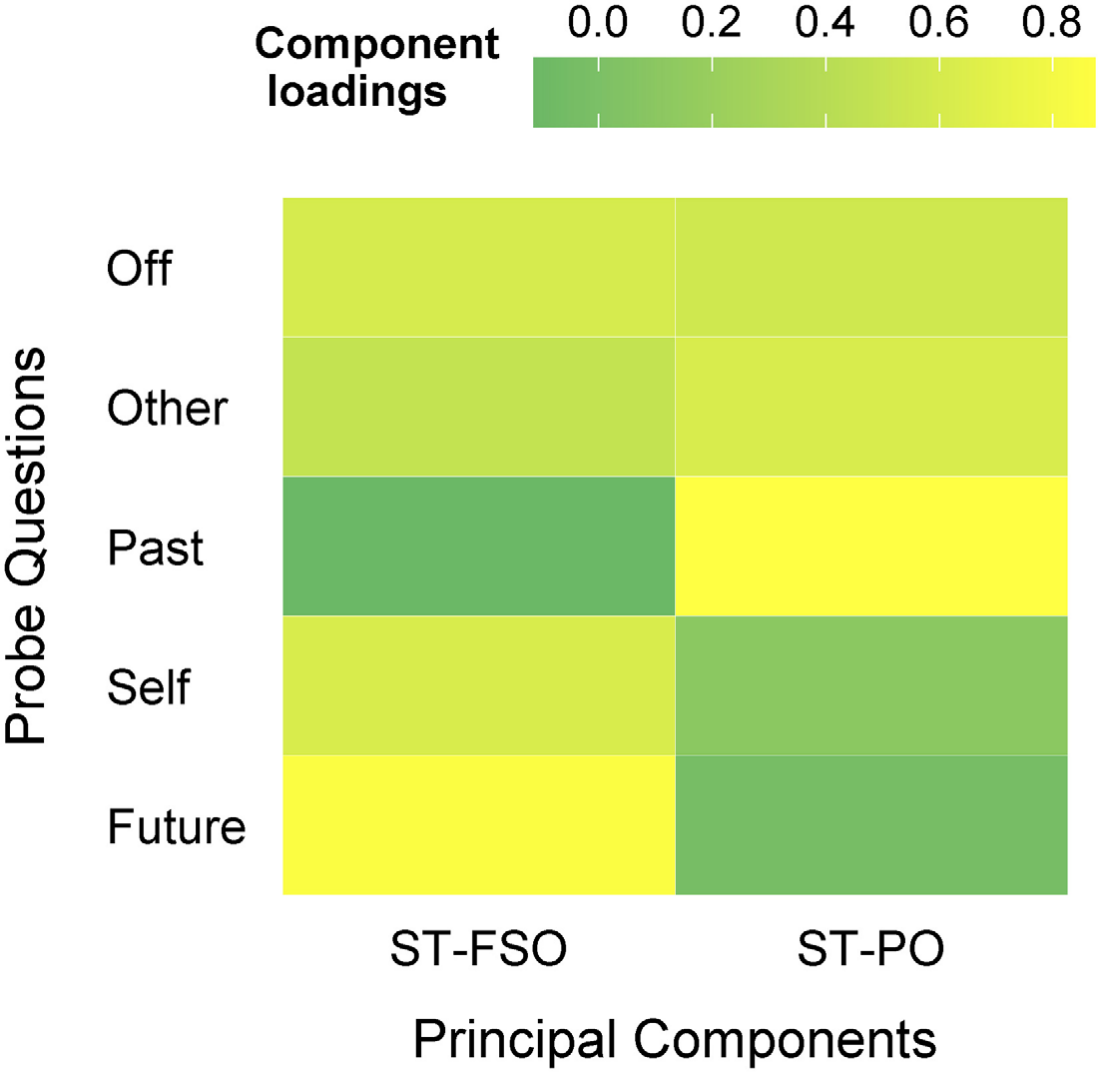
**PCA on SGT ratings revealed two Principal components, one loading positively on Off-task, Future, Self and Other ratings (ST-FSO) and one loading positively on Off-task, Past and Other (ST-PO)**. PCA with Varimax Rotation was applied on 1200 thought probes i.e., regrouping probes from both tasks and all participants.

As it can be seen in Table 2, ratings from different probe questions were highly correlated, suggesting that there is a significant amount of shared variance that should be accounted for. Following Ruby et al. (2013) we used PCA to explore the co-variance between Off-task ratings and measures of SGT content. PCA with Varimax rotation was applied on the Off-task, Future, Past, Self, and Other ratings of 1200 thought probes i.e., regrouping probes from both tasks and all participants. Applying PCA at the thought-probe level allows to take into account both within- and between-subject sources of variance and to compute a measure of the general patterns of SGT that are present in our data. As our variable of interest is SGT, i.e., “mental content that is unrelated to the task in hand” (Smallwood, 2013) the off-task ratings were included in the PCA to appreciate whether the factors obtained were reflecting on-task or off-task thoughts. The Detail ratings were excluded from the PCA to replicate the methods published by Ruby et al. (2013). Two Principal components were obtained that explained 64% of the variance in our sample (Figure 2, Table 1). Both components reflected a combination of social and temporal aspects of SGT: The first socio-temporal component loaded positively on Off-task, Future, Self, and Other ratings (hereafter ST-FSO). The second socio-temporal component loaded positively on Off-task, Past, and Other ratings (hereafter ST-PO). The pattern of co-variance indicates that other-related thoughts are prevalent in both past- and future-related SGT which supports the assumption that both types of SGT may reflect attempts at social problem solving.

**Table 1:** **Component loadings obtained from the PCA with Varimax Rotation on the SGT measures**.

	**ST-FSO**	**ST-PO**
Off	0.602	0.555
Other	0.485	0.617
Past	−0.115	0.875
Self	0.623	0.106
Future	0.852	−0.041

*This information is graphically represented in Figure 2*.

**Figure 3:**
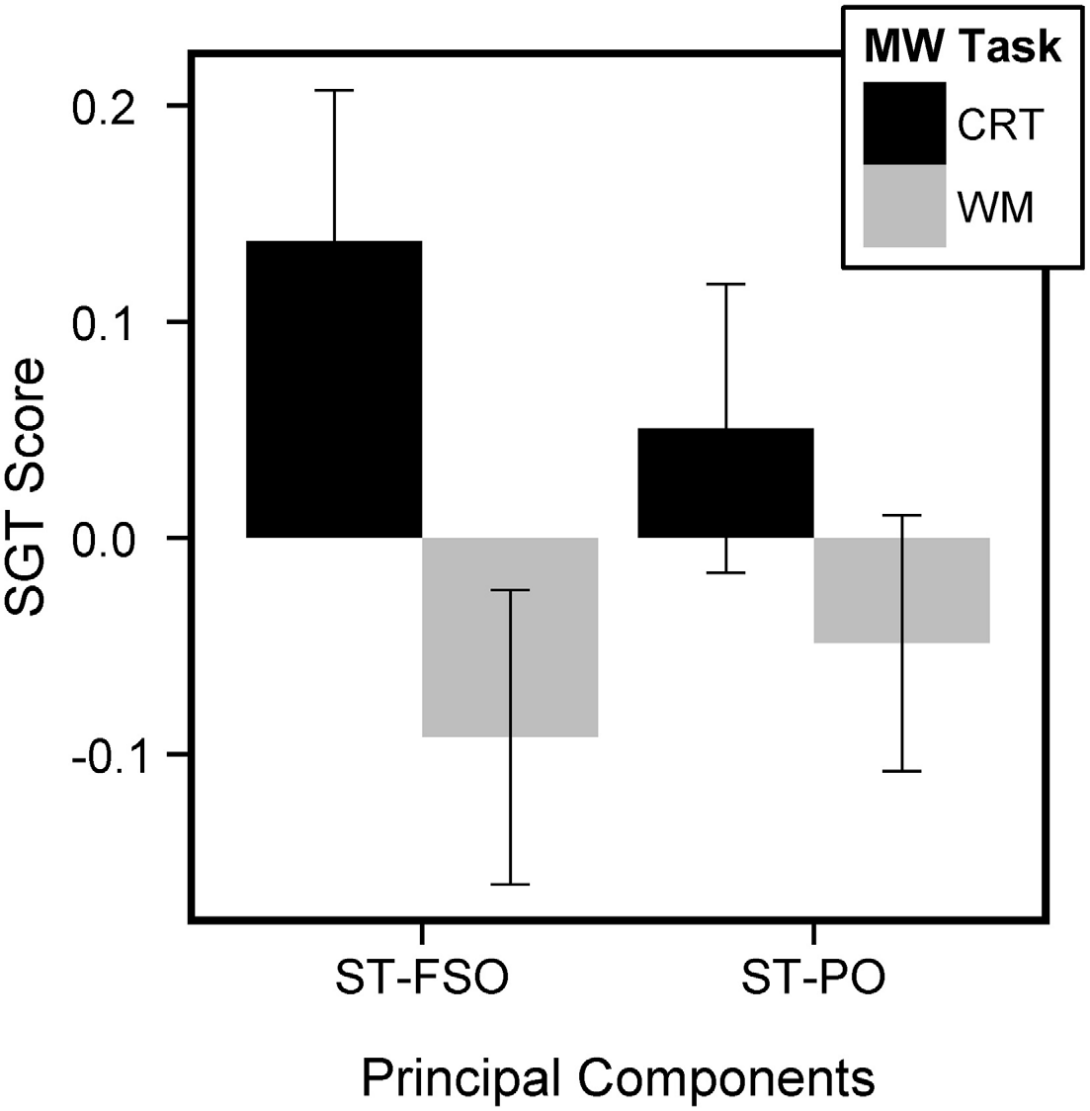
**Mean SGT Scores across subjects for each Principal component and each task**. SGT scores were higher in the CRT than the WM task.

To take into account the possibility that the context in which SGT is measured can have implications for SGT's psychological correlates, we computed 4 SGT scores for each subject for subsequent analyses at the group level: mean ST-FSO in the CRT, mean ST-FSO in the WM, mean ST-PO in the CRT, and mean ST-PO in the WM. We performed a 2-by-2 ANOVA with Task (CRT, WM) and Components (ST-SFO, ST-PO) which revealed a main effect of Task [*F*_(83)_ = 18.57, *p* < 0.001]. This suggests that the amplitude of both components was larger in the CRT than in the WM task, in a similar manner than the ratings they load on (Figure 3). A trend for a Task × Component Interaction [*F*_(83)_ = 3.0, *p* = 0.087] was also observed. For each component, a difference measure was computed between CRT and WM tasks and a paired *t*-test between these measures revealed a trend for a difference across tasks for one component more than the other (*t* = 1.73, *p* = 0.09). Paired *t*-test for each component indicated that the difference observed across tasks may be especially pronounced for ST-FSO (paired *t*-test for ST-FSO, *t* = 4.33, *p* < 0.001; for ST-PO, *t* = 1.84, *p* = 0.07). The results obtained here are very similar to the results obtained from raw probe data: i.e., all probe questions had significantly higher ratings in the CRT vs. the WM task, and future ratings were higher than past ratings, especially in the CRT task. Our PCA approach therefore yields results that are consistent with standard group-level averages and replicates previous findings showing that future-related thinking is higher during non-demanding vs. demanding tasks.

#### Link between SGT and MEPS performance

Table 2 shows Pearson's correlations between probe ratings and MEPS measures. Although informative, raw correlations do not take into account the shared variance between measures (e.g., the relation between MEPS measures) or the nested structure of our data (e.g., within-subject sampling across cognitive tasks). To that end, we favored the use of repeated measures ANOVAs that allow accounting for within- and between-subjects effects. To investigate the link between SGT and MEPS, we first performed a 2-Way repeated measure ANOVA with Task as a single factor (CRT vs. WM), predicting Off-task ratings from MEPS scores. We observed two main effects (Efficiency: *F* = 4.60, *p* < 0.04; RM: *F* = 4.04, *p* < 0.05) but no significant interactions. We therefore computed a mean Off-task score and ran a linear regression with Efficiency and RM as covariates. We observed that an increase in Off-task was negatively correlated with Efficiency (*t* = −2.13, *p* < 0.04) but positively correlated with RM (*t* = 2.0, *p* < 0.05). These results seem to contrast with the raw correlations presented in Table 2. However, the significant effects may emerge when applying the ANOVA because both MEPS measures are included in a single analysis and as a consequence, control for each other. Our results therefore suggest that only the individual variance of each measure (rather than their shared variance) has an opposite relationship with Off-task.

**Table 2:** **Correlation matrix for SGT ratings, task performance and MEPS measures**.

	**Efficiency**	**RM**	**RT**	**Error rate**	**Off-task**	**Detail**	**Self**	**Other**	**Future**	**Past**
**CRT TASK**										
RT	−0.071	−0.089	1.000							
Error rate	−0.069	−0.094	−0.063	1.000						
Off-task	−0.052	0.109	0.002	−0.103	1.000					
Detail	−0.144	−0.261^*^	0.138	−0.076	0.027	1.000				
Self	−0.214^*^	−0.166	−0.019	0.141	0.306^**^	0.107	1.000			
Other	−0.057	0.157	−0.201^+^	−0.162	0.694^***^	0.073	0.250^*^	1.000		
Future	−0.097	0.044	−0.120	−0.155	0.565^***^	0.127	0.436^***^	0.600^***^	1.000	
Past	−0.136	0.042	−0.028	0.123	0.400^***^	0.085	0.338^**^	0.501^**^	0.308^*^	1.000
**WM TASK**										
RT	−0.152	−0.196^+^	1.000							
Error rate	−0.334^**^	−0.168	0.262^*^	1.000						
Off−task	−0.173	0.058	0.012	0.113	1.000					
Detail	−0.265^*^	−0.331^**^	0.242^*^	0.065	−0.066	1.000				
Self	−0.117	−0.103	−0.108	0.072	0.447^***^	−0.035	1.000			
Other	−0.219^*^	0.071	−0.105	0.098	0.702^***^	−0.121	0.266^*^	1.000		
Future	−0.052	0.057	−0.107	0.149	0.472^***^	−0.169	0.379^***^	0.531^***^	1.000	
Past	−0.119	0.103	−0.052	−0.007	0.307^***^	−0.096	0.368^***^	0.274^*^	0.235^*^	1.000

Significance levels are represented with

+ *(p-value < 0.1)*,

* *(p-value < 0.05)*,

** (p-value < 0.01), and

*** (p-value < 0.001).

**Figure 4:**
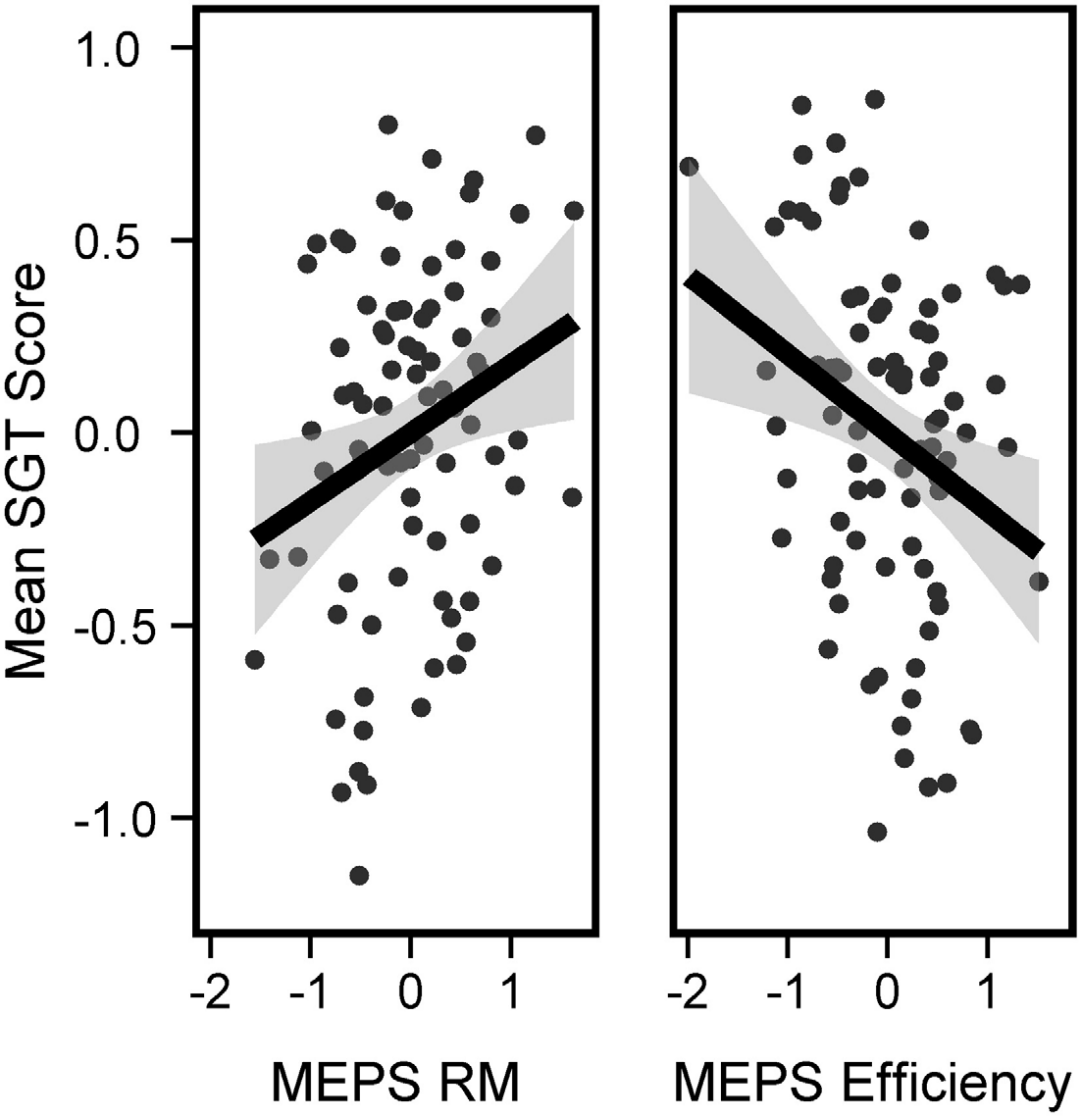
**Link between SGT and MEPS performance**. Mean SGT was associated with higher number of RM (left panel) but lower efficiency (right panel). Mean SGT Score corresponds to the mean of [ST-FSO in CRT, ST-FSO in WM, ST-PO in CRT, and ST-PO in WM] computed for each subject. Black lines represent best-fitted linear regressions and gray areas represent 95% confidence intervals.

To investigate whether the link between Off-task and MEPS varies according to SGT content, we performed a repeated measures 2 × 2 ANOVA (Task: CRT, WM; Components: ST-FSO, ST-PO) predicting SGT with MEPS measures as between-participant covariates. We observed main effects for both Efficiency and RM (Efficiency: *F* = 7.63, *p* = 0.007, RM: *F* = 5.72, *p* = 0.02) but no significant interactions, suggesting that MEPS predicted SGT regardless of content. Separate ANOVAs indicated that both ST-SFO and ST-PO had similar relations to MEPS performance (ANOVA predicting mean ST-PO score from MEPS performance, Efficiency: *t* = −3.19, *p* = 0.002, RM: *t* = 3.07, *p* = 0.003; ANOVA predicting mean ST-SFO score from MEPS Performance, Efficiency: *t* = −1.470, *p* = 0.14, RM: *t* = 1.01, *p* = 0.3). To visualize the results we computed a mean SGT score (mean SGT between ST-SFO and ST-PO, across both tasks) and performed an Univariate ANOVA predicting mean SGT score again with MEPS scores as covariates. Mean SGT score was negatively predicted by Efficiency (*t* = −2.76, *p* = 0.007) but positively predicted by RM (*t* = 2.39, *p* < 0.02, Figure 4). Because we applied the PCA on probe ratings at the trial level, we aim to confirm that this approach did not create artifacts in our results. Therefore, we computed 2 “pseudo-components” by averaging the ratings that characterized ST-FSO and ST-PO (i.e., averaging the probe questions that had a loading coefficient higher than 0.40). This resulted in two pseudo-components: (i) pseudo-FSO (average of off-task, other, future and self ratings) and (ii) pseudo-PO (average of off-task, other and past-ratings). Each pseudo-component was computed separately for each subject and each task, therefore only containing within-subject variance, unlike the group-level PCA components which contain within-subject and between-subject variance. When implementing the 2-by-2 ANOVA with the pseudo-components, we obtained very similar results [Main effect of Efficiency, *F*_(83)_ = 7.07, *p* = 0.009, Main effect of RM, *F*_(83)_ = 5.32, *p* = 0.02]. This confirms that our PCA approach yields results that are consistent with a standard group level average and did not lead to artificial results.

### Link between social problem solving and cognitive tasks performance

#### Link between MEPS and CRT/WM performance

**Figure 5:**
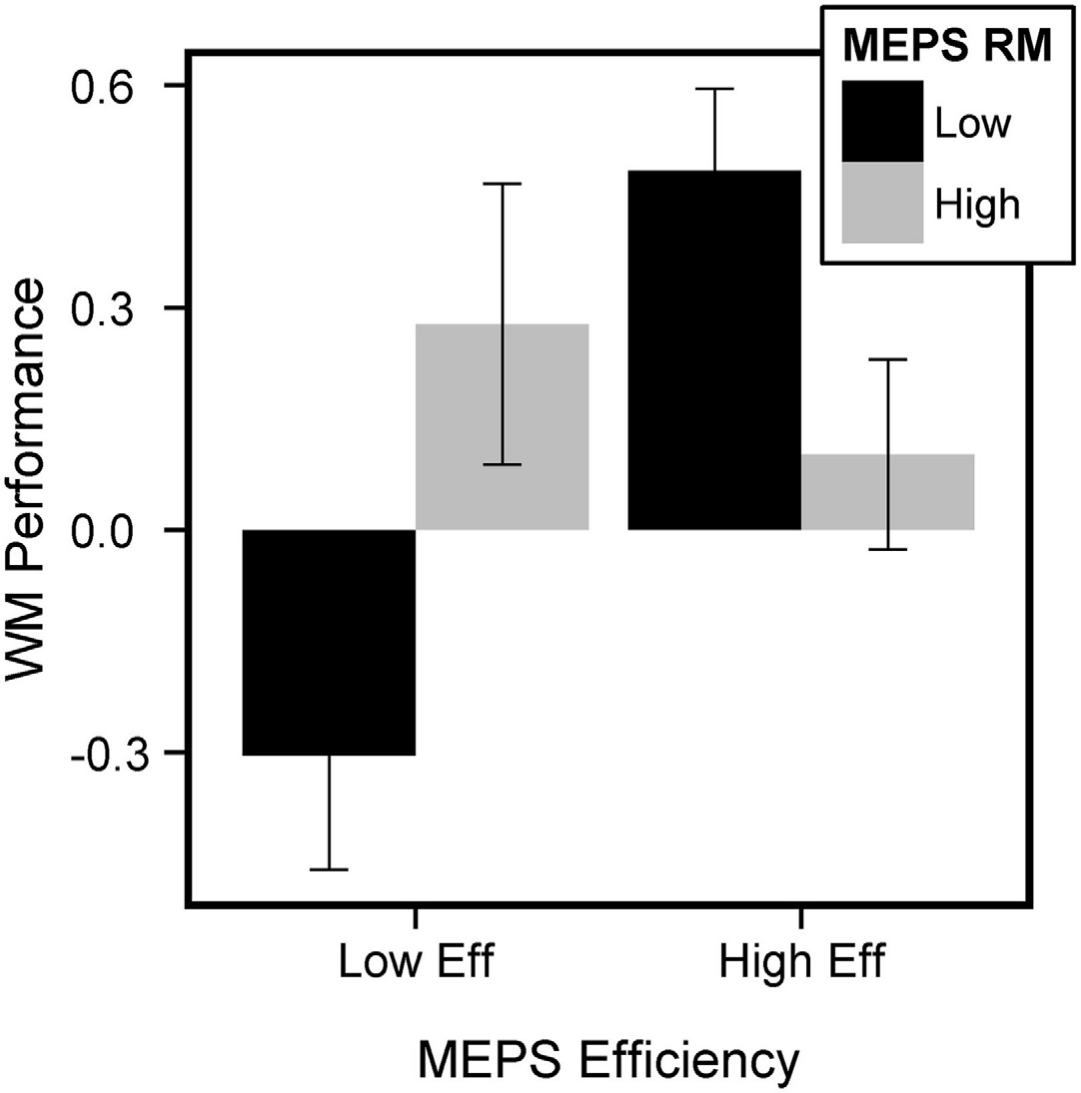
**Link between MEPS and WM performance**. Participants with poor WM performance also had poor MEPS performance (low efficiency, low RM). We performed median splits on Efficiency and RM scores and computed a measure of mean WM performance for each of the 4 groups of participants (both low efficiency and RM, both high efficiency and RM, low efficiency-high RM, high efficiency-low RM). Error bars represent standard error of the mean.

In addition to the link between SGT and social problem solving, our data also allowed us to look at the relationship between the MEPS and cognitive task performance i.e., whether efficient problem solvers were also good at performing the CRT and WM tasks. Pearson's correlations between MEPS measures and task performance are reported in Table 2. We computed a measure of task performance, by averaging Error Rate and RT (previously z-scored) and reversing the score. This was calculated separately for the CRT and WM task. We computed a repeated-measures 2-way ANOVA (Task: CRT, WM) predicting task performance with Efficiency and RM as covariates. This analysis revealed a trend for a triple interaction (Task × Efficiency × RM, *F* = 3.52, *p* = 0.06) but no significant main effects (Efficiency, *F* = 0.67, *p* > 0.4; RM, *F* = 0.84, *p* > 0.3). Separate ANOVAs for each task indicated that the interaction between Efficiency and RM was almost significant in the WM task (*t* = −1.9, *p* = 0.06) but not in the CRT task (*t* = 0.21, *p* > 0.8). To visualize the interaction, we performed median splits on both Efficiency and RM and plotted these against WM task performance. As can be seen on Figure 5, WM performance was particularly poor when both Efficiency and RM were low. Although our result is only at trend level, it suggests that participants with poor performance at the WM task, but not the CRT, also performed poorly on the MEPS.

#### Shared variance between SGT and cognitive task performance

As both WM task performance and SGT significantly predicted the MEPS, we investigated whether these two measures explained shared or separate MEPS variance. We conducted a repeated measures 2-Way ANOVA predicting MEPS Efficiency and RM, with WM task performance and mean WM SGT score as covariates. Both measures still significantly predicted MEPS performance (Main effect of WM performance, *F* = 7.32, *p* = 0.008; WM mean SGT × MEPS interaction, *F* = 11.05, *p* = 0.001). Similarly to the previous analysis, WM performance was associated with poor overall MEPS (Univariate ANOVA predicting mean MEPS performance, main effect of WM task performance: *t* = 2.81, *p* = 0.006). SGT was associated with increased RM and reduced Efficiency (Univariate ANOVA for RM controlling for Efficiency, mean WM SGT: *t* = 2.81, *p* = 0.006; Univariate ANOVA for Efficiency controlling for RM, mean WM SGT: *t* = −3.34, *p* = 0.001). This suggests that both WM performance and SGT explain distinct features of MEPS variance.

### Link between SGT and cognitive tasks performance

**Figure 6:**
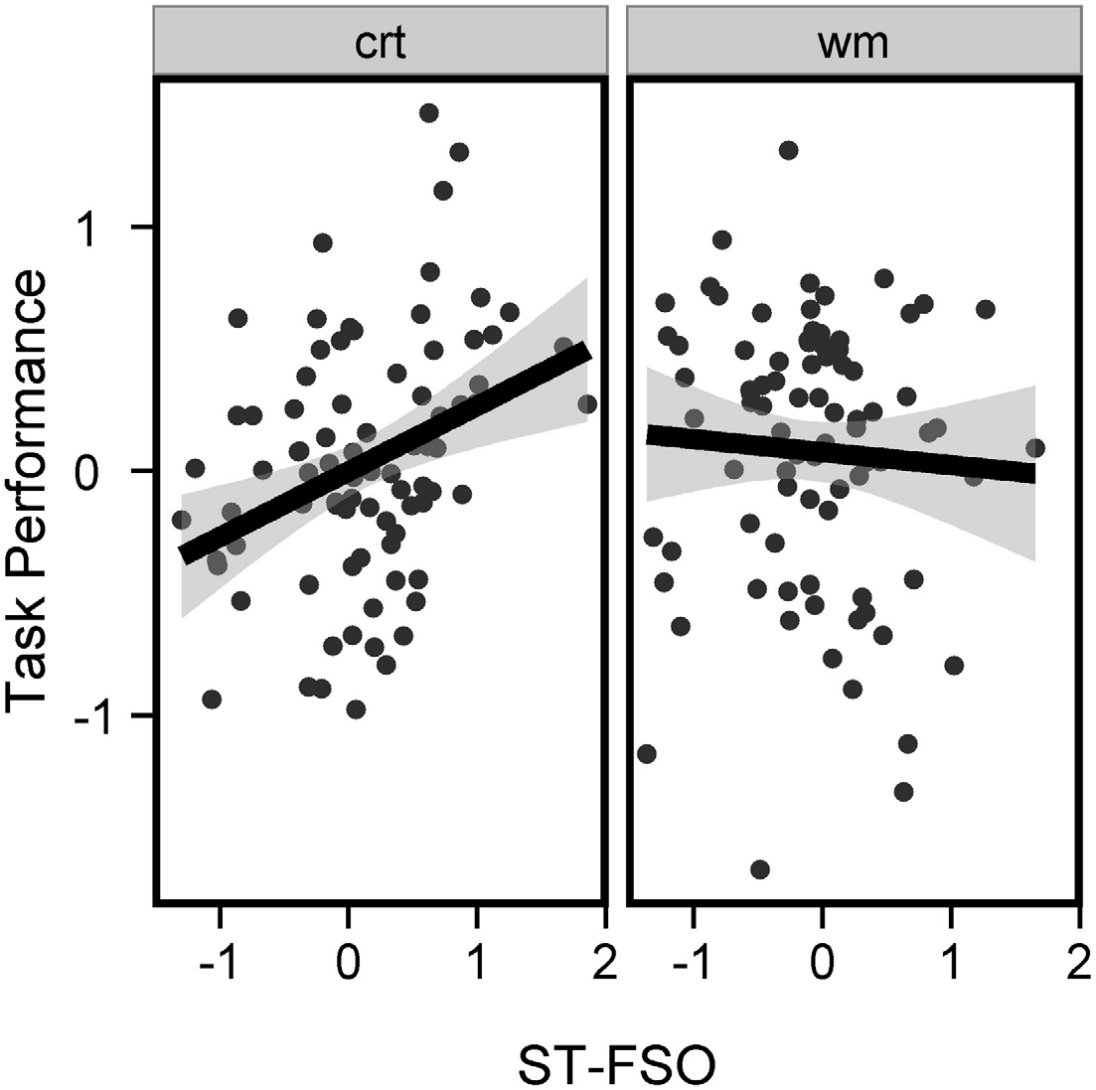
**Link between SGT and cognitive tasks performance**. Task performance was plotted against the ST-FSO Score for each subject, separately for each cognitive task. Increase in ST-FSO was associated with increased performance during the CRT task (left panel, *p* < 0.07) but had no significant effect in the WM task (right panel). Black lines represent best-fitted linear regressions and gray areas represent 95% confidence intervals.

Finally, we investigated the relation between SGT and the cognitive tasks being performed. To account for the nested structure of our data, we used a linear mixed model (Bates et al., 2012) in order to predict performance on each task based on SGT. *p*-values were estimated using the *pvals.fnc* function provided in the languageR package (Baayen, 2008). ST-PO, ST-FSO, and Task (CRT, WM) were defined as fixed effects and Subject as a random effect. We observed a significant positive effect of ST-FSO on performance (*t* = 1.99, *p* < 0.05) whereas ST-PO had no significant effect (*t* = −0.59, *p* > 0.5). In addition, an interaction between Task and ST-FSO was also observed at trend level (*t* = −1.88, *p* = 0.06). ANOVAs performed separately on each task revealed a trend for an effect of ST-FSO on CRT Performance (*t* = 1.84, *p* < 0.07) but not on WM performance (*t* = −0.62, *p* > 0.5, Figure 6). This suggests that an increase in SGT directed toward the future, and involving oneself and others, may be associated with better task performance, especially during the CRT task. Again, we confirmed that these resulted were not artifacts caused by our PCA by performing the linear mixed model again using the pseudo-components calculated before. The results were again very similar (Main effect of pseudo-FSO, *t* = 2.13, *p* = 0.02; Main Interaction between Task and pseudo-FSO, *t* = −2.01, *p* = 0.04; Effect of pseudo-PO, *t* = −1.72, *p* = 0.09; Task × pseudo-PO × pseudo-FSO interaction, *t* = −1.79, *p* = 0.08).

## Discussion

This study sets out to explore the relationship between social problem solving and SGT. We were interested in examining whether general SGT levels or specific types of SGT are linked to social problem-solving abilities. To define different types of SGT, we decomposed our thought-sampling data using PCA at the trial level, which revealed two components that where differentiated by temporal focus (Past vs. Future) and both loaded strongly on other-related thoughts. This pattern of co-variance suggests that regardless of temporal focus, SGT often involves thoughts about other people and so is consistent with the general premise that many of these experiences may reflect attempts at solving social problems. When investigating the link between SGT and MEPS, we observed that increases in SGT levels, regardless of the content, were linked to reduced efficiency but increases in RM. Our findings therefore do not support the hypothesis that different types of SGT may have different links to social problem solving. It is possible that the absence of differentiation between types of SGT reflects idiosyncratic features of our sample, or a failure to operationalize the content of SGT in an appropriate manner. However, as we replicated the finding that future-related thinking was higher in the non-demanding CRT vs. WM task, our German sample is broadly similar to groups studied in a number of different countries (Smallwood et al., 2009; Andrews-Hanna et al., 2010; Baird et al., 2011; Iijima and Tanno, 2012; Song and Wang, 2012). Moreover, our finding that ST-FSO (but not ST-PO) varied with task demands and was associated with better performance on the CRT task demonstrates that our different measures of content are operationalized sufficiently well as to be able to differentiate between experimental manipulations and objective-dependent measures. It seems reasonable to assume that our failure to find evidence of a clear differentiation between specific content of SGT and social problem solving cannot therefore be explained by unique features of our population or from a failure to operationalize the content of SGT. Importantly, we obtained very similar findings when using pseudo-components computed at the group level, therefore demonstrating that our results did not arise because of a possible artifact caused by our PCA approach (see the Limitations section for a complete discussion of this issue).

Although it remains a possibility that the association between SGT and social problem solving may depend on the content of thought if we had operationalized our questions differently, it is also possible that our results illustrate a general relationship between SGT and social problem solving. Plausibly SGT and social problem solving share a common dependence on the capacity to generate mental content that is based on memory rather than a direct representation of reality as it is now. This is consistent with accounts of SGT that emphasize that the unique features of such experiences is the motivated generation of mental content that are distinct from perception (Smallwood, 2013) and with formulations of problem solving which emphasize the importance of generative thinking in the production of novel solution steps. It is also consistent with prior work showing that individuals who engage in daydreaming (as assessed via a retrospective measure) generate more solutions to creative problems (Baird et al., 2012). More generally, the positive association between SGT and RM is consistent with the *Current Concerns Hypothesis* proposed by Klinger (1978, 1999). According to this hypothesis, cognition is often devoted to events that are salient to individuals, and when the external environment lacks sufficiently compelling input, individuals engage in SGT to provide a source of stimulation. As the *Current Concerns Hypothesis* assumes that the motivating feature for SGT is an attempt to resolve personal concerns, it elegantly captures our finding that individuals who habitually engage in SGT generate more RM in order to solve the MEPS problems.

Not only was SGT associated with more RM, it was also associated with a reduction in the effectiveness of the solutions, as judged by our independent raters. One interpretation of this result is that the relation between SGT and social problem solving may take the form of a Yerkes-Dodson relationship, with optimal problem solving being achieved by individuals who display a suitable balance between SGT and cognition derived from perceptual input. Plausibly, there may be three different populations of problem-solvers: one who generates low levels of SGT and produces short but efficient solutions to problems, a second who produces large numbers of solutions with reasonable levels of efficiency and a third who generates the most steps but whose solutions are ineffective. Although speculative, this interpretation of our data is consistent with evidence that SGT can have both costs and benefits to tasks performance (for a review, see Smallwood, 2013). Moreover, it suggests a reason why interventions that cultivate mindfulness and which reduce the tendency for mind-wandering (Mrazek et al., 2012b, 2013) may be beneficial in daily life. By restraining the mind's habitual tendency to wander, interventions that emphasize being in the moment may help individuals gain a degree of control over SGT which may in turn allow them to employ this basic capacity for conscious thought to generate solutions in a more efficient manner.

We also found that participants with poor problem solving abilities (characterized by both low RM and Efficiency) performed poorly on a moderately demanding WM task when controlling for SGT. As the WM task relies on greater controlled processing than does the CRT, this suggests that the capacity to deploy controlled processing to an external task and to social problem solving relies on similar cognitive processes (such as working memory or attentional resources). This is consistent with prior studies that have demonstrated that individuals high on measures of controlled processing are also good problem solvers, as well as fMRI investigations which link domain general processes of control to the solution of both spatial and autobiographical problems (Spreng et al., 2010). Finally, a positive link between SGT and task performance was in particular observed during the non-demanding CRT task and may reflect the finding that future-related thought in the CRT is linked to higher working memory (Baird et al., 2011). Our result of better performance associated with ST-FSO is in contrast to other studies that find that in general, experience unrelated to a task elicits a negative influence on task performance (for a review, see Smallwood, 2011). This result suggests that future studies understanding the negative influence of SGT on task performance should take in account both task context as well as the content of thoughts (Smallwood and Andrews-Hanna, 2013).

## Limitations

The overarching framework guiding the current study is the hypothesis that meaningful psychological information is contained in the variation in SGT content across situations and individuals (Smallwood and Andrews-Hanna, 2013). As a consequence, we sought to define categories of SGT that have the potential to simultaneously vary within and between participants. We applied unconstrained PCA at the trial level and examined how the resultant components varied in a quantitative fashion across individuals and situations. This novel approach allowed us to investigate three types of psychological relationships to SGT: (i) possible within-subjects effects on the SGT content (e.g., “Does SGT vary depending on the task?”), (ii) between-subject effects (e.g., “Does SGT content vary according to MEPS performance?”), and (iii) their interaction (e.g., “Do certain individuals exhibit different patterns of thoughts in different contexts?”). Given the novelty of our data analysis strategy, it is worth explicitly considering its strengths and weaknesses.

One practical question is whether our approach yields results that are either unreliable or inconsistent with what is known about SGT: this would provide straightforward evidence that this approach lacks utility. In terms of replicability, the PCA components we obtained in the current study have a striking resemblance to previously published SGT components obtained from an independent dataset (Ruby et al., 2013). This consistency across datasets suggests that the components obtained from an unconstrained trial-level PCA are reasonably replicable. Our work using the trial-by-trial PCA also found results consistent with previous findings in the SGT literature. In terms of within-participant variance, ST-FSO was higher in a non-demanding task: this is consistent with a prospective bias to SGT found by several different laboratories (Smallwood et al., 2009; Andrews-Hanna et al., 2010; Baird et al., 2011; Iijima and Tanno, 2012; Song and Wang, 2012). Our PCA approach also yielded group-level effects that have been demonstrated by prior studies. Ruby and colleagues reported that ST-PO is linked to higher BDI score (Ruby et al., 2013), an observation that is consistent with the increased retrospective focus found in dysphoria and unhappiness (Smallwood and O'Connor, 2011; Poerio et al., 2013; Stawarczyk et al., 2013). The application of PCA at the trial level therefore captures both within and between-participant variation consistent with other methods of analysis. Altogether this suggests that trial level PCA can yield reliable results that are both replicable and are consistent with prior work.

There are also theoretical questions regarding how to best decompose SGT in order to characterize the psychological correlates of the within and between-participant variance in thought content [for a review see (Smallwood, 2013; Smallwood and Andrews-Hanna, 2013)]. Dimension reduction techniques can be employed that control for both within and between-subject variance during data decomposition e.g., multi-level exploratory factor analysis (Reise et al., 2005). These provide a more generalizable description of the thoughts of the population because they seek covariance that is common across the sample (or to a particular context). However, these techniques make categorizing variation *across* individuals, or contexts, more difficult, because by optimizing the decomposition process to seek commonality, differences across people or context are a feature of the unexplained variance. As we are interested in describing the psychological significance of the co-variance between and within participants, we did not want to constrain the decomposition process using this information. Instead, we tested the psychological features of within and between-participant variance derived from our unconstrained PCA by including the individual and context as factors in subsequent analyses. Our demonstration that ST-FSO was higher in the CRT task (i.e., within-participant variance) for individuals who performed the task especially well (i.e., between-participant variance, see Figure 6) indicates that we were successful in this regard. Although the specific features of SGT that we find using trial-by-trial PCA may not generalize to other samples, this result demonstrates that co-variance across both tasks and participants provide meaningful psychological information regarding the content of thought (Smallwood, 2013; Smallwood and Andrews-Hanna, 2013). A second alternative would be to employ PCA *separately* to each task at the group level; this would allow an investigation of qualitative differences in PCA structure in different contexts; however, it would make it impossible to quantify how specific patterns of thought change across situations because the PCA components calculated in this fashion would not be directly comparable. While the possibility remains that the application of PCA at the trial level could lead to unrepresentative results, the available data suggests that the technique yields results that are (i) reliable across independent data sets, (ii) provides valid accounts of existing phenomena, (iii) captures psychological differences that are contained in the patterns of covariance in multiple reports of SGT, and (iv) allows the quantitative capture of both within and between-subject co-variation in different aspects of SGT. We suggest that future research should continue to use this technique in an exploratory fashion because of its potential value in mapping the heterogeneity of SGT that arises through the combination of the constraints placed by different environmental situations and the range of individual differences that contribute to the content of thought.

There are a number of further limitations that should be borne in mind when considering our data. Concerning our methodological approach, we only administered a measure of *social* problem solving and it remains to be seen whether the link between problem solving and control processes or SGT is specific to the social domain or may generalize to other forms of problem resolution. In addition, although problems were chosen to mimic daily-life concerns, they remain hypothetical. Anderson et al. (2009) compared measures of problem solving abilities obtained from the MEPS and from a “Real-Life problem solving diary task,” in which participants' reported a problem when it occurs and describe afterwards the strategy they used to solve it. Although they found that the performance at both tasks could predict future depressive symptoms, they also observed that both measures explained different portions of variance and did not correlate. This suggests that, although the MEPS remains an important tool as it measures the ability to generate responses to problems under laboratory conditions, Anderson notes that “it is the successful implementation of such strategies that ultimately impacts upon the situation's outcome” [p. 54 (Anderson et al., 2009)]. Although our data suggests that individuals reporting more SGT may also have improved problem solving skills, it does not allow us to draw conclusions regarding their actual success in daily life. Our data is also correlational and though our results confirm links between social problem solving and SGT, we cannot identify whether fluency at social problem causes SGT or vice versa, nor can we specify the mechanism that links these two phenomena without further study. Based on studies showing a positive relation between MEPS performance and autobiographical memory retrieval [e.g., (Evans et al., 1992; Goddard et al., 1996, 1997)] and the link between SGT and autobiographical memory (Baird et al., 2011; Smallwood, 2013), the association between SGT and social problem solving may emerge through their mutual requirement of autobiographical memory. One important future direction would therefore be to assess memory capacities or to use neuroimaging methods to determine the specific cognitive processes that mediate the link between social problem solving skills and SGT [e.g., (Spreng et al., 2010)]. Given that social problem solving is important to the success of every member of society, understanding the mechanism that links this experience to states of perceptually-guided thought and particularly SGT are recommended.

### Conflict of interest statement

The authors declare that the research was conducted in the absence of any commercial or financial relationships that could be construed as a potential conflict of interest.

## References

[B1] Anderson R. J. Goddard L. Powell J. H. (2009). Social problem-solving and depressive symptom vulnerability: the importance of real-life problem-solving performance. Cognit. Ther. Res. 35, 48–56 10.1007/s10608-009-9286-2

[B2] Andrews-Hanna J. R. Reidler J. S. Huang C. Buckner R. L. (2010). Evidence for the default network's role in spontaneous cognition. J. Neurophysiol. 104, 322–335 10.1152/jn.00830.2009 20463201 PMC2904225

[B3] Baayen R. H. (2008). languageR: Data Sets And Functions With “Analyzing Linguistic Data: A Practical Introduction to Statistics.” R Packag. Version 0.953. Available online at: http://cran.r-project.org/web/packages/languageR/

[B4] Baird B. Smallwood J. Mrazek M. D. Kam J. W. Franklin M. S. Schooler J. W. (2012). Inspired by distraction: mind wandering facilitates creative incubation. Psychol. Sci. 23, 1117–1122 10.1177/0956797612446024 22941876

[B5] Baird B. Smallwood J. Schooler J. W. (2011). Back to the future: autobiographical planning and the functionality of mind-wandering. Conscious. Cogn. 20, 1604–1611 10.1016/j.concog.2011.08.007 21917482

[B6] Bates D. Maechler M. Bolker B. (2012). lme4: Linear Mixed-Effects Models Using S4 Classes. Available online at: http://cran.r-project.org/package=lme4

[B7] Christoff K. Gordon A. M. Smallwood J. Smith R. Schooler J. W. (2009). Experience sampling during fMRI reveals default network and executive system contributions to mind wandering. Proc. Natl. Acad. Sci. U.S.A. 106, 8719–8724 10.1073/pnas.0900234106 19433790 PMC2689035

[B8] D'Zurilla T. J. Goldfried M. R. (1971). Problem solving and behavior modification. J. Abnorm. Psychol. 78, 107–126 10.1037/h003136 4938262

[B9] Evans J. Williams J. M. G. Oloughlin S. Howells K. (1992). Autobiographical memory and problem-solving strategies of parasuicide patients. Psychol. Med. 22, 399–405 10.1017/S0033291700030348 1615107

[B10] Frith C. D. Frith U. (2006). The neural basis of mentalizing. Neuron 50, 531–534 10.1016/j.neuron.2006.05.001 16701204

[B11] Goddard L. Dritschel B. Burton A. (1996). Role of autobiographical memory in social problem solving and depression. J. Abnorm. Psychol. 105, 609–616 10.1037/0021-843X.105.4.609 8952194

[B12] Goddard L. Dritschel B. Burton A. (1997). Social problem solving and autobiographical memory in non-clinical depression. Br. J. Clin. Psychol. 36(Pt 3), 449–451 10.1111/j.2044-8260.1997.tb01252.x 9309360

[B13] Iijima Y. Tanno Y. (2012). [The effect of cognitive load on the temporal focus of mind wandering]. Shinrigaku Kenkyu 83, 232–236 10.4992/jjpsy.83.232 23012825

[B14] Klinger E. (1978). Dimensions of thought and imagery in normal waking states. J. Altered States Conscious. 4, 97–113

[B15] Klinger E. (1999). Thought flow: properties and mechanisms underlying shifts in content, in At Play in The Fields of Consciousness: Essays in Honor of Jerome L. Singer, ed Salovey J. A. S. P. (Mahwah, NJ: Lawrence Erlbaum Associates Publishers), 29–50

[B16] Lyubomirsky S. Nolenhoeksema S. (1995). Effects of self-focused rumination on negative thinking and interpersonal problem-solving. J. Pers. Soc. Psychol. 69, 176–190 10.1037/0022-3514.69.1.176 7643299

[B17] Marx E. M. Williams J. M. G. Claridge G. C. (1992). Depression and social-problem solving. J. Abnorm. Psychol. 101, 78–86 10.1037/0021-843X.101.1.78 1537977

[B18] Mrazek M. D. Franklin M. S. Phillips D. T. Baird B. Schooler J. W. (2013). Mindfulness training improves working memory capacity and GRE performance while reducing mind wandering. Psychol. Sci. 24, 776–781 10.1177/0956797612459659 23538911

[B19] Mrazek M. D. Smallwood J. Franklin M. S. Chin J. M. Baird B. Schooler J. W. (2012a). The role of mind-wandering in measurements of general aptitude. J. Exp. Psychol. 141, 788–798 10.1037/a0027968 22468669

[B20] Mrazek M. D. Smallwood J. Schooler J. W. (2012b). Mindfulness and mind-wandering: finding convergence through opposing constructs. Emotion 12, 442–448 10.1037/a0026678 22309719

[B21] Platt J. J. Spivack G. (1975). Manual for the Mean-end Problem-solving Procedure (MEPS): A Measure of Interpersonal Cognitive Problem-solving Skill. Hahnemann Community Mental Health/Mental Retardation Center, Philadelphia, PA: Hahnemann Medical College and Hospital

[B22] Poerio G. L. Totterdell P. Miles E. (2013). Mind-wandering and negative mood: does one thing really lead to another? Conscious. Cogn. 22, 1412–1421 10.1016/j.concog.2013.09.012 24149091

[B23] R Core Team. (2012). R: A Language and Environment for Statistical Computing. Available online at: http://www.r-project.org

[B24] Reise S. P. Ventura J. Nuechterlein K. H. Kim K. H. (2005). An illustration of multilevel factor analysis. J. Pers. Assess. 84, 126–136 10.1207/s15327752jpa8402_02 15799887

[B25] Ruby F. J. M. Smallwood J. Engen H. Singer T. (2013). How self-generated thought shapes mood—the relation between mind-wandering and mood depends on the socio-temporal content of thoughts. PLoS ONE 8:e77554 10.1371/journal.pone.0077554 24194889 PMC3806791

[B26] Schneider W. Eschman A. Zuccolotto A. (2002). E-Prime User's Guide. Pittsburgh, PA: Psychology Software Tools Inc

[B27] Smallwood J. (2011). Mind-wandering while reading: attentional decoupling, mindless reading and the cascade model of inattention. Lang. Linguist. Compass 5, 63–77 10.1111/j.1749-818X.2010.00263.x

[B28] Smallwood J. (2013). Distinguishing how from why the mind wanders: a process-occurrence framework for self-generated mental activity. Psychol. Bull. 139, 519–535 10.1037/a0030010 23607430

[B29] Smallwood J. Andrews-Hanna J. (2013). Not all minds that wander are lost: the importance of a balanced perspective on the mind-wandering state towards a balanced perspective of the mind-wandering state. Front. Psychol. 4:441 10.3389/fpsyg.2013.00441 23966961 PMC3744871

[B30] Smallwood J. Brown K. S. Tipper C. Giesbrecht B. Franklin M. S. Mrazek M. D. (2011a). Pupillometric evidence for the decoupling of attention from perceptual input during offline thought. PLoS ONE 6:e18298 10.1371/journal.pone.0018298 21464969 PMC3064669

[B31] Smallwood J. Schooler J. W. Turk D. J. Cunningham S. J. Burns P. Macrae C. N. (2011b). Self-reflection and the temporal focus of the wandering mind. Conscious. Cogn. 20, 1120–1126 10.1016/j.concog.2010.12.017 21277803

[B32] Smallwood J. Nind L. O'Connor R. C. (2009). When is your head at? An exploration of the factors associated with the temporal focus of the wandering mind. Conscious. Cogn. 18, 118–125 10.1016/j.concog.2008.11.004 19121953

[B33] Smallwood J. O'Connor R. C. (2011). Imprisoned by the past: unhappy moods lead to a retrospective bias to mind wandering. Cogn. Emot. 25, 1481–1490 10.1080/02699931.2010.545263 21432633

[B34] Smallwood J. Ruby F. J. M. Singer T. (2013). Letting go of the present: mind-wandering is associated with reduced delay discounting. Conscious. Cogn. 22, 1–7 10.1016/j.concog.2012.10.007 23178292

[B35] Smallwood J. Tipper C. Brown K. Baird B. Engen H. Michaels J. R. (2012). Escaping the here and now: evidence for a role of the default mode network in perceptually decoupled thought. Neuroimage 1428, 60–70 10.1016/j.neuroimage.2012.12.012 23261640

[B36] Song X. Wang X. (2012). Mind wandering in Chinese daily lives–an experience sampling study. PLoS ONE 7:e44423 10.1371/journal.pone.0044423 22957071 PMC3434139

[B37] Spreng R. N. Stevens W. D. Chamberlain J. P. Gilmore A. W. Schacter D. L. (2010). Default network activity, coupled with the frontoparietal control network, supports goal-directed cognition. Neuroimage 53, 303–317 10.1016/j.neuroimage.2010.06.016 20600998 PMC2914129

[B38] Stawarczyk D. Majerus S. D'Argembeau A. (2013). Concern-induced negative affect is associated with the occurrence and content of mind-wandering. Conscious. Cogn. 22, 442–448 10.1016/j.concog.2013.01.012 23466878

[B39] Svaldi J. Dorn C. Trentowska M. (2011). Effectiveness for interpersonal problem-solving is reduced in women with binge eating disorder. Eur. Eat. Disord. Rev. 19, 331–341 10.1002/erv.1050 20957769

[B40] Watkins E. Baracaia S. (2002). Rumination and social problem-solving in depression. Behav. Res. Ther. 40, 1179–1189 10.1016/S0005-7967(01)00098-5 12375726

[B41] Wickham H. (2009). Ggplot2: Elegant Graphics for Data Analysis. New York, NY: Springer Available online at: http://bvbr.bib-bvb.de:8991/F?func=service&doclibrary=BVB01&docnumber=017387312&linenumber=0001&funccode=DB_RECORDS&servicetype=MEDIA 10.1007/978-0-387-98141-3

